# Substrate recognition by two different P450s: Evidence for conserved roles in a common fold

**DOI:** 10.1038/s41598-017-14011-w

**Published:** 2017-10-19

**Authors:** Drew R. Tietz, Allison M. Colthart, Susan Sondej Pochapsky, Thomas C. Pochapsky

**Affiliations:** 1 Department of Chemistry, Brandeis University, MS 015, 415 South St, Waltham, MA 02453 USA; 2 Department of Biochemistry and the Rosenstiel Basic Medical Sciences Research Institute, Brandeis University, 415 South St., Waltham, MA 02453 USA

## Abstract

Cytochrome P450 monooxygenases CYP101A1 and MycG catalyze regio- and stereospecific oxidations of their respective substrates, *d*-camphor and mycinamicin IV. Despite the low sequence homology between the two enzymes (29% identity) and differences in size and hydrophobicity of their substrates, the conformational changes that occur upon substrate binding in both enzymes as determined by solution NMR methods show some striking similarities. Many of the same secondary structural features in both enzymes are perturbed, suggesting the existence of a common mechanism for substrate binding and recognition in the P450 superfamily.

## Introduction

Cytochrome P450 monooxygenases typically activate molecular oxygen, enabling O_2_ to react with otherwise unactivated C-H and C-C bonds in their substrates. These oxidations can be highly regio- and stereoselective, and as such, P450s are often found in the biosynthetic pathways leading to complex bioactive molecules (steroid hormones, antibiotics, toxins) or in the breakdown of compounds that can be used as sources of carbon and reducing equivalents. The cytochrome P450 superfamily is vast, with nearly 500,000 entries in GenBank ascribed to P450s as of early 2017, and members of the superfamily having been characterized for all kingdoms and nearly all phyla of life. Although many of these enzymes likely perform identical functions in different organisms, they nevertheless represent a vast array of potential substrate-product combinations, with substrates ranging in size from monoterpenes to macrocyclic and polycyclic compounds that exhibit a wide range of functionality and hydrophobicity.

It is remarkable that despite this diversity, the P450 superfamily exhibits a unique and highly conserved fold, with identical topology and (usually) conserved secondary structural features identified in all of the P450 structures determined to date^1^. Clearly, the P450 fold was arrived upon early in evolution as a safe platform for an inherently dangerous reaction, the activation of molecular oxygen^2^. This dichotomy presents some fascinating questions: What structural or dynamic features must be conserved for the enzyme to safely activate O_2_? What can be modified in order to fill a particular metabolic niche? Once a substrate is recognized and bound, how does the enzyme orient substrate appropriately for the observed chemistry? Given this combination of functional diversity and structural/mechanistic conservatism, the P450 superfamily provides an excellent platform for understanding the role of structural and sequence variability in a conserved enzyme function.

**Figure 1:**
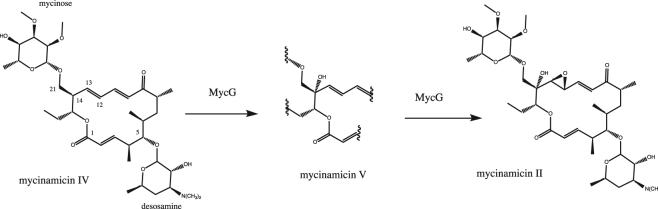
Monooxygenase activities of MycG.

We have found that nuclear magnetic resonance (NMR) can provide satisfying answers to some of the questions raised above. While X-ray crystallography is the most efficient means of obtaining structural information for P450s, relating the crystal structure to mechanism is not always straightforward. Substrate orientations may not rationalize the observed chemistry^3^, and it is not a given where on the reaction pathway the enzyme conformation that crystallizes may lie, or even if it is mechanistically relevant. Solution NMR permits tight control of conditions (e.g., oxidation state, ligands/substrates, ionic strength) so that the relevance of structure and dynamics to a given point in the reaction pathway can be more readily assessed^1^. Chemical shift perturbation and dynamics measurements provide insight into those structural features that are involved in substrate and effector recognition^4–8^, and residual dipolar couplings (RDCs) can be used to characterize enzyme conformations that are populated as a function of substrate or effector binding^9–13^. We have previously used RDC-directed “soft annealing” molecular dynamics simulations to show that CYP101A1 (cytochrome P450_cam_) undergoes significant conformational changes upon binding of substrate *d*-camphor^11,12^, and via site-directed mutagenesis coupled with activity assays, demonstrated that the observed changes are not artifacts of the methodology, but reliable representations of the conformational equilibria accessed by CYP101A1 in the presence and absence of substrate^10^. We recently extended this methodology to generate solution conformations of cytochrome P450 MycG with substrate mycinamicin IV bound^9^. MycG catalyzes the last two steps of mycinamicin II biosynthesis in the bacterium *Micromonospora griseorubida*, a regiospecific hydroxylation followed by epoxidation of an adjacent C = C double bond in the macrolactone ring (Fig. 1) ^3,14^. In the current work, we use RDCs to characterize the solution conformers accessed by MycG in the absence of substrate, and compare the conformational changes that occur upon substrate binding in both enzymes, MycG and CYP101A1, in order to identify functional similarities between two P450s with different sequences, substrates and biological roles.

## Results

The methodology used to generate the solution structures discussed here has been detailed in previous publications^9,11,12^. In brief, sequential resonance assignments for backbone amide ^1^H, ^15^N correlations in MycG were made using multidimensional NMR data sets obtained with a combination of uniformly and type-selective ^2^H, ^13^C and ^15^N labeled samples. With these assignments in hand, samples of ^15^N-labeled enzyme are mixed with an alignment medium, that is, an additive that exhibits a significant magnetic susceptibility anisotropy. In a magnetic field, these additives align so as to minimize their interactions with the field (assuming the additives are diamagnetic). To a first approximation, globular proteins tumble isotropically in solution, averaging the dipolar coupling between nuclear spins (such as that between ^1^H and ^15^N in an amide bond) to zero. However, in the presence of an alignment medium, the protein molecules acquire a degree of anisotropy in their motions, re-introducing the dipolar coupling in a highly attenuated fashion (hence the name residual dipolar couplings, or RDCs)^15^. The sign and magnitude of the RDC for a given N-H correlation can be measured as a modulation of the field-independent scalar J-coupling^16^. In turn, these RDCs can be used as restraints in molecular simulations, as their signs and magnitudes are determined by the orientations of the N-H bond vectors they represent with respect to an alignment tensor. The structures thus generated are free of crystal packing constraints, and given that RDCs can be measured for different sets of conditions (e.g., with or without substrate, different oxidation states of the heme, different ligands), the response of the structure to changing conditions can be ascertained.

**Figure 2:**
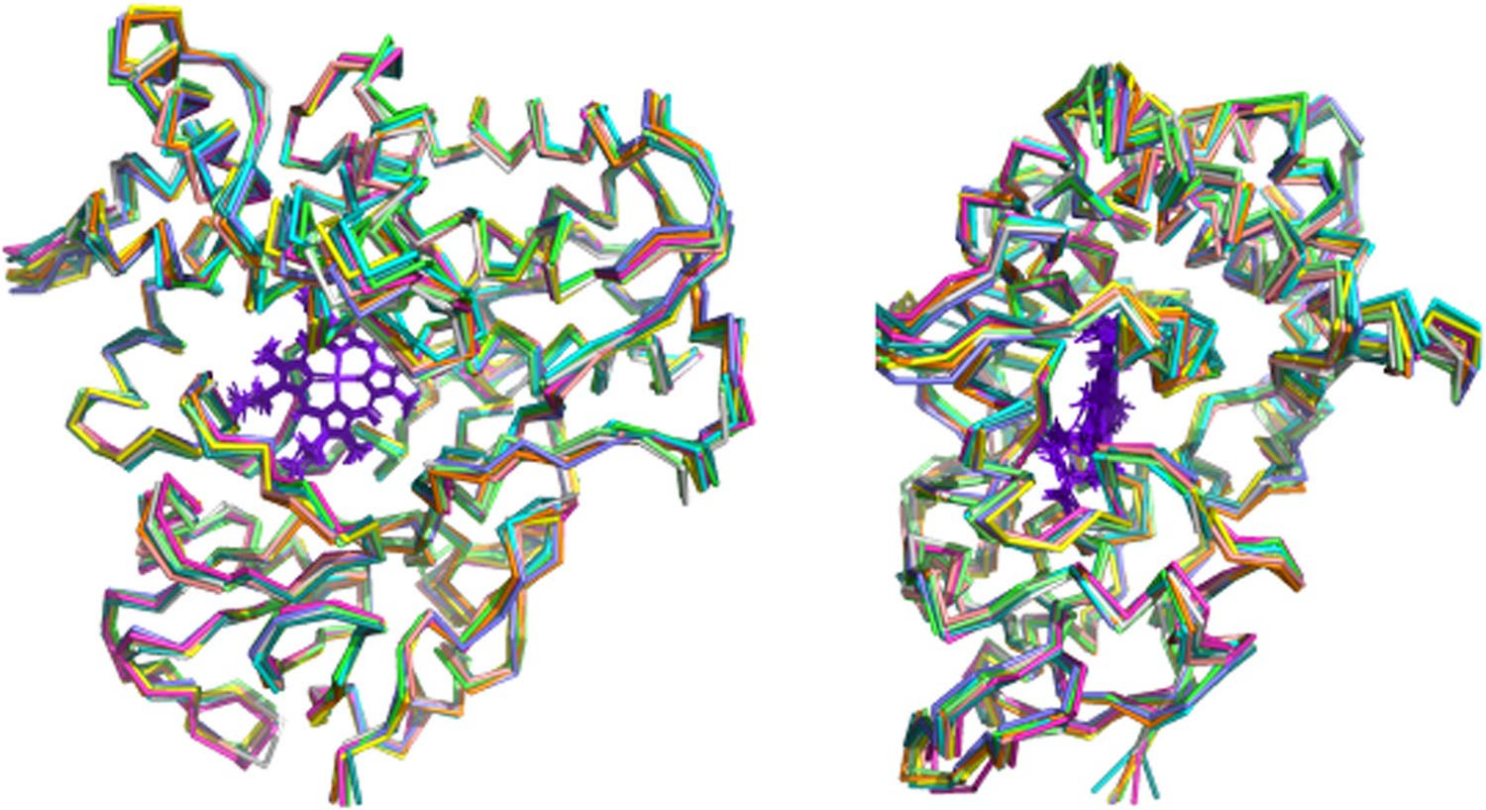
Superposition of ten lowest RDC violation energy structures of substrate-free MycG. Figure at left is in the same orientation as REP2 in Fig. 3, and the figure at right is rotated counterclockwise around the vertical by 90°. Figure generated using PyMOL^22^.

**Figure 3:**
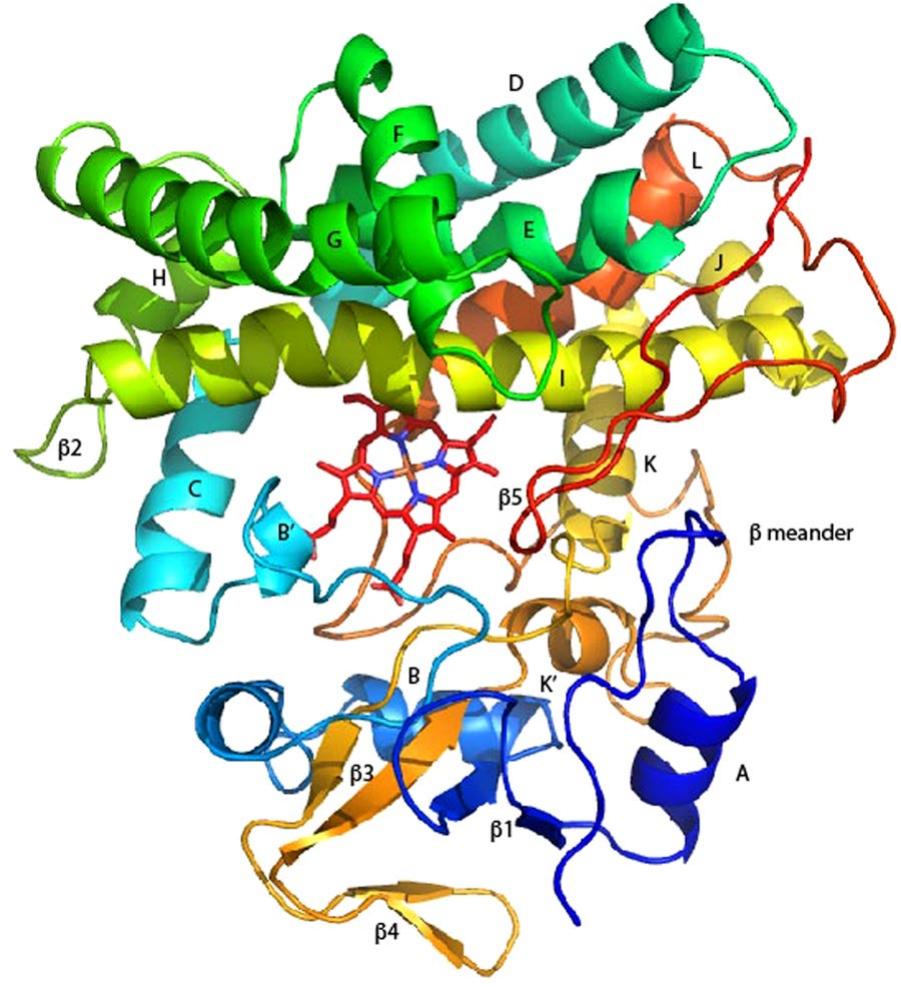
RDC-restrained solution structure of substrate-free MycG, REP2. Secondary structures are labeled according to the scheme of Poulos^20^. Color progresses from blue (N-term) to red (C-term). Heme is shown in red sticks near the center of the structure. Helix (A), Gly 17-Thr 25; **β1** (strand 1) Val 28-Val 31, (strand 2) Ala 39-Val 42; (B), Tyr 45-Gly 53; (B**’**), Pro 72-Lys 76; (C), Pro 85-Ala 97; (D), Ala 100-Thr 124; (E) Asp 129-Leu 147; (F), His 154-Ala 163; (G), Ala 172-Lys 195; (H), Leu 201-Gln 207; **β2** (strand 1), Arg 209 and Asp 210, (strand 2), Asp 213 and Ser 214; (I) helix, Glu 217-Thr 248; (J), Pro 250-Asp 258; (K), Pro 260-Trp 273; **β3** (strand 1), Arg 284-Ala 286; **β4** (strand 1) Val 290-Leu 292, (strand 2) Val 295-Ile 297; **β3** (strand 2), Gly 300-Ala 305; (K’), Thr 307-Asn 311; (L), Ala 345-Arg 362, **β5** (strand 1), Leu 375-Gly 380, (strand 2) Arg 384-Pro 386). Sequence numbering is (-4) relative to that of the crystal structure of M-IV-bound MycG (PDB entry 2Y98) as the first four residues were not included in the current calculations.

In the current work, we measured RDCs for substrate-free MycG in two alignment media, *pf1* bacteriophage and a nematic liquid crystal phase, C_12_E_5_/*n*-hexanol^17^, and used the measured RDCs as restraints in a “soft annealing” molecular dynamics protocol described previously^9^. The RDC-generated structure for mycinamicin IV (M-IV)-bound MycG (PDB entry 1UHU) was used as a starting point for the calculations, all of which were performed on the XSEDE node Comet (UCSD) using the Amber 16 software suite^18^. After removing bound M-IV, the structure was solvated with 19014 discrete water molecules and neutralized with sufficient K^+^ and Cl^−^ ions to match the ionic strength used in the NMR measurements (0.2 M). The heme iron was retained as hexacoordinate 3 + (ferric) with the crystallographically confirmed axial water ligand retained. After an initial minimization, the simulation was heated to 300 K in 60000 2 ps steps and equilibrated at constant volume for 200 ps, followed by 1 ns of equilibration at constant pressure. Once a constant density of 1.012 kg/L was reached, RDC restraints were applied with a small energy penalty (0.001 kcal mol^−1^ ΔD^−1^, where ΔD is the difference between measured and calculated RDCs outside of +/−3 Hz uncertainty). The simulations were performed in short increments of 200 ps in order to identify incorrectly measured or ambiguously assigned RDCs and optimize alignment tensor components prior to beginning production runs. The energy penalty for RDCs was gradually increased over the course of the optimization runs to a final value of 0.01 kcal mol^−1^ ΔD^−1^, which we have previously found to provide sufficient restraint for structural analysis without distortion of local bond geometries^12^. After ~6 ns of simulation, we arrived at a refined set of 234 RDCs measured in *pf1* media, and 221 RDCs from C_12_E_5_ (Tables S2 and S3). At this point, we began to select those structures with the lowest RDC violation energies for further analysis (Fig. 2). With relatively little variation between the lowest-energy structures, we selected that closest to the average, obtained at 7.552 ns, as a representative of the ensemble, REP2, shown in Fig. 3.

Unlike the calculations with CYP101A1 and M-IV-bound MycG (all of which reached a plateau without repeat RDC violations after the iterative correction process described above), a significant number of unambiguously assigned and measured RDCs in both RDC data sets (ten RDCs from *pf1* and eleven from C_12_E_5_, Table S1) were found to give consistent violations for substrate-free MycG. To understand this observation, we performed a series of simulations in which *only* those RDCs that gave rise to significant energy penalties were used, in order to determine whether these would have a marked effect on the structures generated. A low-violation structure generated in this fashion is superimposed on REP2 in Figure S1, with the locations of those residues producing the violations indicated. While the alignment tensors generated using these RDCs are markedly different from those that were optimized in the global calculations, the overall disposition of structural features does not change significantly between the two structures. Most of the RDCs that exhibit deviations are located on external structural features and, and are almost always larger in magnitude than those predicted from the alignment tensors generated during the global calculations (Tables S1, S4 and Fig. S1). This observation leads us to suspect that the deviations are due to local interactions between the alignment media and solvent-exposed regions of the structure, resulting in increased local alignment.

It is important to remember that the measured RDCs represent time averages of instantaneous dipolar couplings, and would average to zero in isotropic media. As such, the RDC-restrained structures described here and elsewhere represent best fits of the experimental restraints from a conformational search over many thermally accessible conformations. In turn, these structures represent averaged positions for secondary structural features, and many other conformations, some more open and some more compact, are accessed both *in silico* and *in vivo*.

### Global conformational changes upon binding of M-IV to MycG

**Figure 4:**
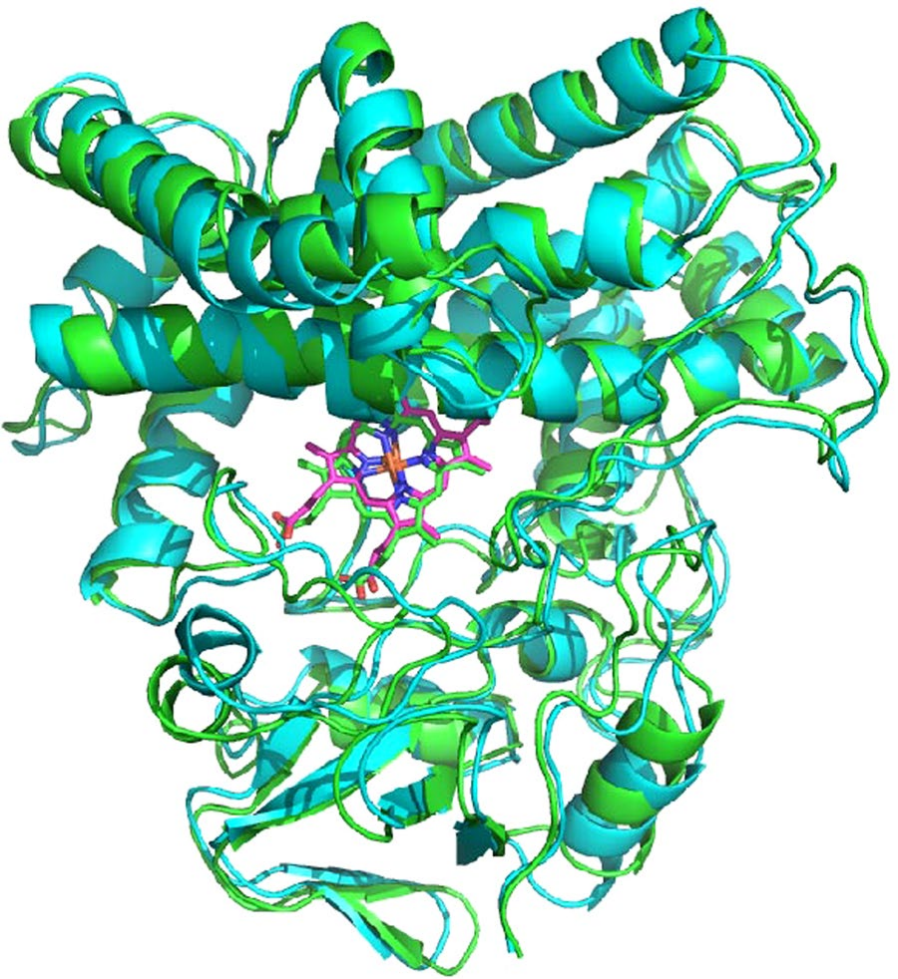
Superposition of solution structures of substrate-bound MycG (green, PDB entry 4UHU) on substrate-free MycG (cyan, REP2). Orientations are the same as shown in Fig. 3. Superposition obtained using Cα carbons, and shows an RMSD of 1.472 Å.

Figure 4 shows a superposition of our recently described solution structure of MycG with substrate M-IV bound (green, PDB entry 5UHU)^9^ upon the current substrate-free structure REP2 (cyan). Attached videos (Video S1 through S6), generated using Chimera^19^, animate the superposition in order to highlight specific features of the observed substrate-dependent conformational displacements to be discussed. Note that all secondary structural features are conserved in both structures, with no noticeable fraying of helices or other structural motifs in the substrate-free enzyme.

Video S1 provides a general view of the conformational changes that occur upon substrate binding. The largest displacements occur in the A, F and G helices. A counterclockwise rotation of the entire β-rich region (the lower portion of the structure as viewed in Fig. 3) moves the edge of the β3 sheet into contact with bound substrate. The movement of the A helix is coupled to this rotation, and accounts for its large displacement.

Video S2 provides a closer look at the rotation of the β-rich region: The pivot axis appears to be close to Thr 43, which lies on the turn between the β1 sheet and B helix. The motion is accompanied by a distortion of the short K’ helix (Ser 306 to Asp 313), with several intra-helical main chain hydrogen bonds changing length substantially in the course of the movement (Video S3). These observations are consistent with the relatively large substrate-dependent chemical shift perturbations observed for ^15^N- ^1^H correlations in the K’ helix noted previously^9^, and appear to reflect a pronounced preference for a 3,10 helical hydrogen bonding pattern (*i, i* + 3 C = O—HN) between Ala 310 and Asp 313 in the presence of substrate, while the hydrogen bonds between Thr 306-Ala 310 and Thr 307-Asn 311 are stable in a standard α-helical hydrogen bonding pattern (*i, i* + 4 C = O—HN) in both forms.

### Active site perturbations

The structural features immediately adjacent to the active site of MycG include the F and G helices, which form an active site “cap” in all canonical P450 structures, and the I helix, a primary contact for the heme and substrates. Both the F and G helices are displaced upon substrate binding, with the G helix being displaced the most: Indeed, it appears that the G helix motion is coupled to the counterclockwise rotation of the β-rich region described above, pivoting around the contact between the I and G helices, and forcing the F helix down further into the substrate binding pocket (Videos S1 and S4). The movements of the I helix are more subtle in terms of overall displacement, but more dramatic upon closer inspection. As shown in Video S5, the I helix has a distinctive “kink” or bend near the heme iron. This “kink” is a common feature of P450 structures, and was first noted by Poulos *et al*. in the first high-resolution crystal structure of a P450, that of P450cam (CYP101A1)^20^.

Those workers proposed that the kink, formed by an interruption in the normal helical hydrogen bonding pattern in the I helix, provided a niche for the binding of O_2_ to the heme iron. Upon binding of substrate to MycG, the kink (formed by the absence of a typical *i, i* + 4 hydrogen bond between the carbonyl oxygen of Ala 230 and the amide NH of Ser 234) is displaced by a rotation of the N-terminal portion of the I helix around the helix long axis (Videos S5 and S6). This rotation breaks the hydrogen bond interaction between the Leu 228 carbonyl oxygen and the NH of Gly 231 while introducing a relatively long hydrogen bond between Ala 230 and Ser 234.

## Discussion

**Figure 5:**
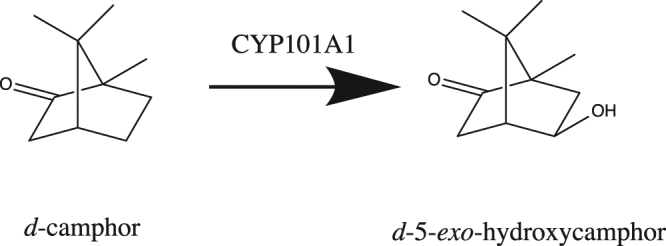
Monooxygenase activity of CYP101A1.

MycG now provides a second example of substrate-dependent conformational changes in a cytochrome P450 as informed by RDCs, and it is of interest to compare these results with those published previously for the camphor hydroxylase CYP101A1, cytochrome P450_cam_^10–12^. Unlike MycG, CYP101A1 is monofunctional: it catalyzes the stereospecific hydroxylation of camphor at the 5-*exo* position (Fig. 5). Sequence identity between the two enzymes is low, with BLAST alignment yielding 29% identity.

**Figure 6:**
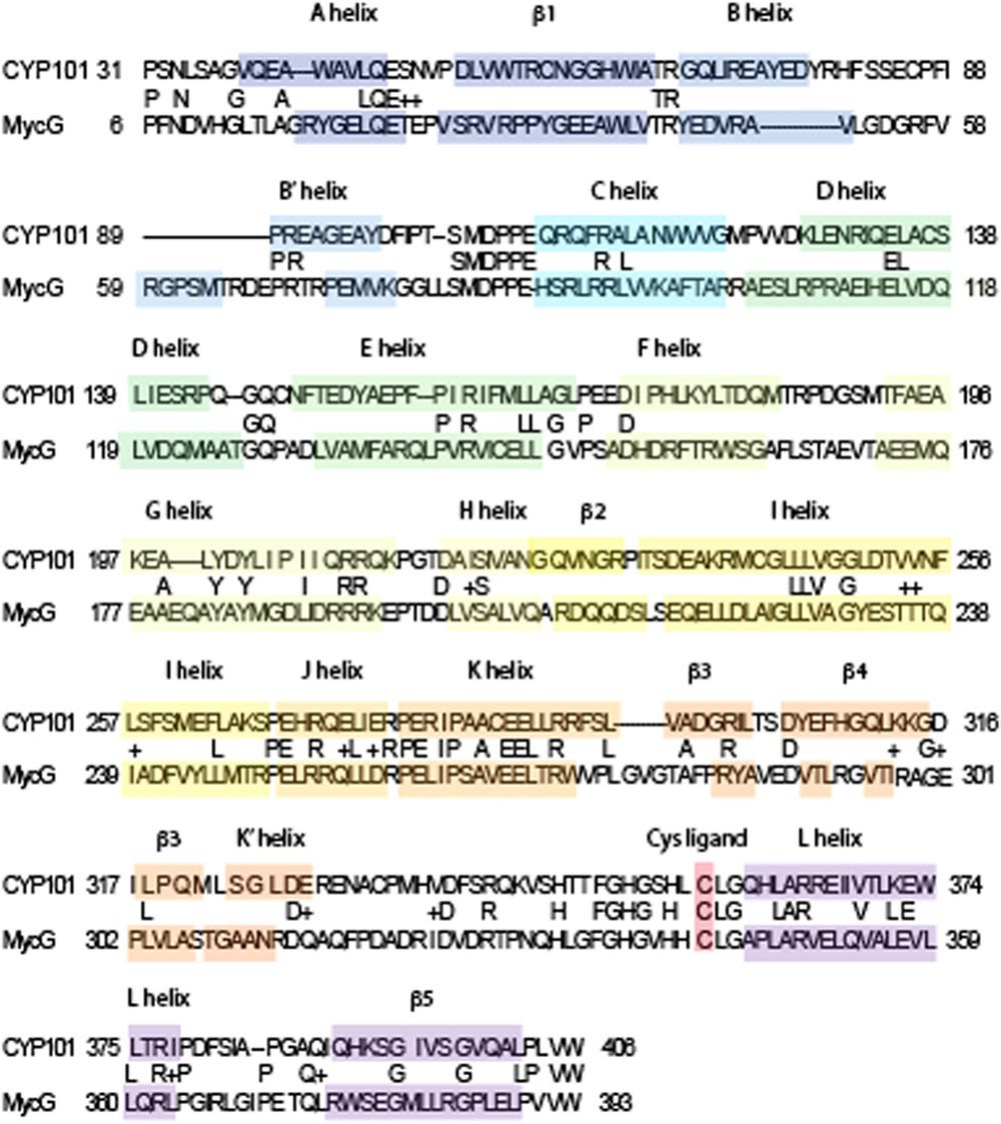
Sequential and structural alignment of CYP101A1 and MycG. Corresponding secondary structural features are highlighted in the same color, with name of the feature corresponding to Fig. 3. Identities and conservations are noted between the sequences.

**Figure 7:**
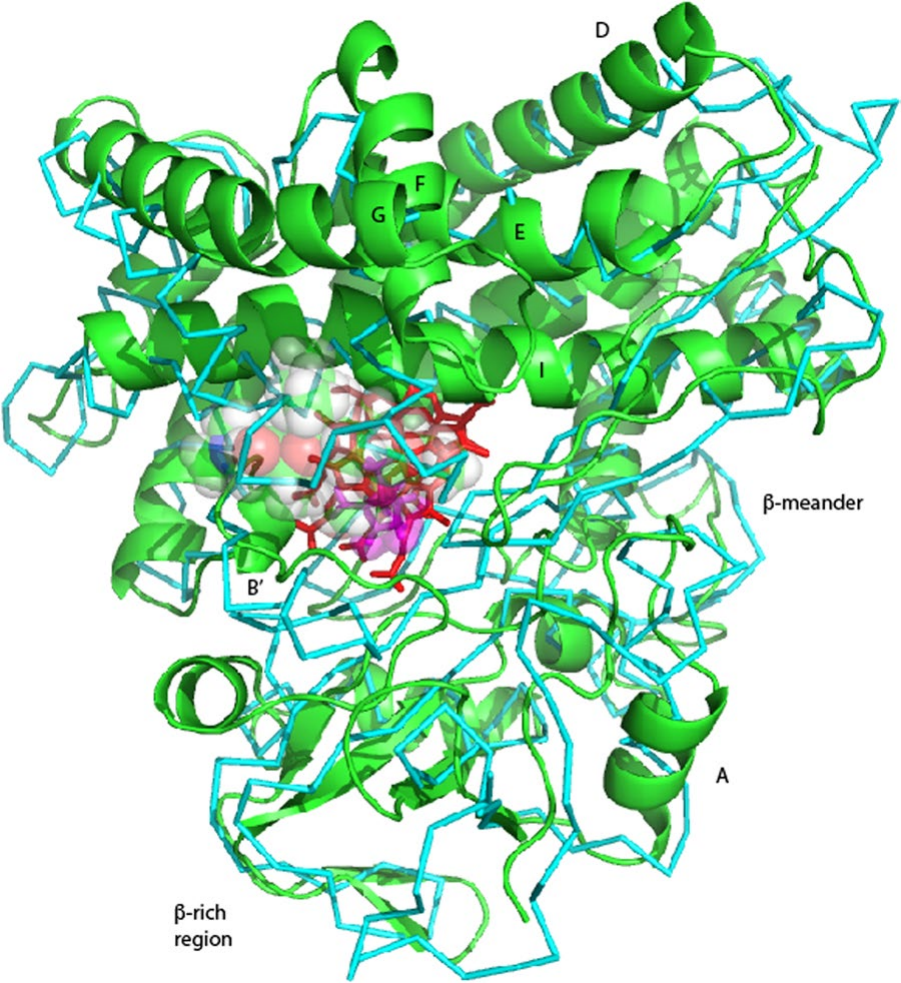
Superposition of substrate-bound CYP101A1 (2L8M, in cyan, backbone connections) with M-IV bound MycG (5UHU, in green, as ribbon structure). Best fit of Cα atom positions give an RMSD of 3.02 Å. Atoms of both substrates are shown as translucent spheres (M-IV light colored, camphor in purple). Heme is shown as red sticks.

Furthermore, structural alignment (i.e., identical residues at corresponding points in the structure alignment) yields an even lower identity, 23.9%. Figure 6 compares sequential and structural homology matching between the two enzymes, while Fig. 7 provides a best-fit superposition of the substrate-bound forms of the two enzymes. Not surprisingly, regions that are involved in direct substrate contacts show the most variability: The B helix in MycG is shorter by ~two turns than in CYP101A1, but a small additional helix in Myc G (Arg 59-Met 64) forms part of what is the B-B’ loop in CYP101A1. The B’ helix is also shorter and less well defined in MycG than in CYP101A1. In both enzymes, these features are involved in substrate placement and orientation, the B’ helix by direct substrate contacts and the B helix by positioning of the B-B’ loop. The β3 sheet, which provides substrate contacts and forms one edge of the active site in both enzymes, is also shorter and less well-defined in MycG than in CYP101A1. All of these observations are compatible with the fact that the active site must be larger and more flexible in MycG than in CYP101A1 in order to accommodate a much larger substrate.

Despite these differences, a comparison of the conformational shifts that occur in both enzymes upon substrate binding yields some striking similarities. In both cases, the β-rich region comprised of the β1, β3 and β4 sheets as well as the A, B, and K’ helices move as a unit to accommodate substrate (Videos S1 and S7). As in CYP101A1^5^, the K’ helix of MycG appears to be a critical component of this motion, acting as a “spring” to position substrate contact Phe 282 near the beginning of the β3 strand by changing hydrogen bonding patterns to favor alternatively a longer, narrower 3,10 helix versus a shorter α-helix (Video S3). The other end of the β3 strand is anchored at a constant distance from the heme by a bidentate salt bridge between one heme propionate and the guanidinium group of the conserved Arg 284. In CYP101A1, Arg 299 serves the same function, while Val 295 is the substrate contact residue whose position near the beginning of the β3 strand is also adjusted by the K’ helix^5^.

### Active site changes upon substrate binding

In CYP101A1, we noted that, in the absence of substrate, the active site is largely collapsed, with Phe 87 on the B-B’ loop moving in to occupy much of the space occupied by camphor in the bound form. Along with these, residues on the B’ and I helices and the loop between the β5 strands rearrange so as to minimize the volume of the active site cavity (See Video S9). In MycG, similar displacements occur: Arg 71 and Glu 73 on the B-B’ loop move to occupy some of the space vacated by substrate MI-V, as do B’ residues Gly 77-Leu 79, I helix residues Ile 225 and Val 229 and β5 loop residues Leu 382 and 383 (Video S3). Because of the difference in size of the active site cavities in the two enzymes, these displacements are more pronounced in MycG than in CYP101A1.

### I helix and associated displacements

In both MycG and CYP101A1, the binding of substrate is accompanied by a rotation of the N-terminal half of the I helix around the helical axis. These rotations result in changes in the hydrogen bonding patterns near the O_2_-binding site “kink” in both enzymes (Videos S5 and S10). However, the sense of the rotation in each case differs, and the perturbations that occur elsewhere as a result differ as well. In CYP101A1, the rotation is counter-clockwise, and result in changes in the interactions between sterically hindered residues Ile 160 on the E helix and Leu 250 and Val 254 on the I helix^10^. These changes are transmitted mechanically via the E helix to the C-D loop (residues Val 123 and Val 124), which in turn show the largest substrate-dependent displacements of any portion of the CYP101A1 molecule (Video S7)^10^. Mutations in this region of CYP101A1 have a profound effect on function, including changes in enzyme efficiency, selectivity, stability and substrate-dependent spin state shifts^10^.

In MycG, the sense of the I helix rotation is clockwise, and multiple steric interactions between I helix residues and residues in the F and G helices dominate the mechanical coupling. These include Leu 221 (I) with Tyr 183 (G), Ile 225 (I) with Phe 164 (F) and Tyr 232 (I) with Leu 165 on the F-G loop. These movements are coupled to the displacements of the F and G helices discussed above, and appear to help “clamp” the substrate M-IV in the active site. In CYP101A1, there is little displacement of the F and G helices upon substrate binding.

### The β-meander regions

Between the C-terminal end of the K’ helix and the axial Cys thiolate ligand for the heme lies a region of irregular structure often referred to as the “β-meander”. The meander is sufficiently stabilized by steric interactions and interresidue hydrogen bonds that it is displaced as a unit in both MycG and CYP101A1 upon substrate binding. However, the displacement is much larger relative to other structural features in MycG than in CYP101A1 (see Videos S1 and S7).

## Conclusions

**Figure 8:**
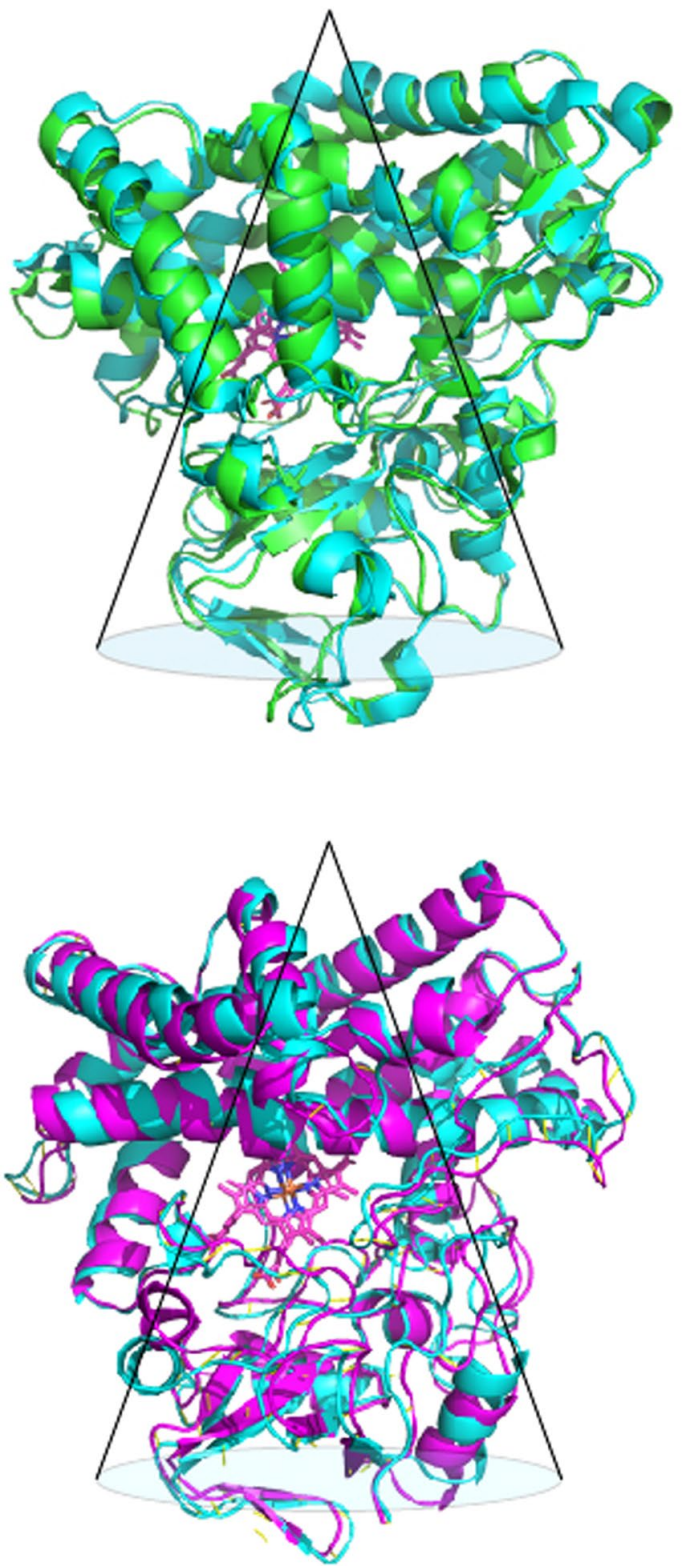
(Top) Superposition of substrate-free (cyan) and substrate-bound (green) CYP101A1. Regions of greatest displacement appear to be encompassed by the conical region shown. See text for details. (Bottom) Superposition of substrate-free (violet) and substrate-bound (cyan) MycG. Again, the largest displacements appear to encompassed by an antisymmetric conical region. See text for details.

Our recent publications have shown via a combination of NMR structural studies, site directed mutagenesis and functional assays that regions well removed from the active site of CYP101A1 are critical to the efficient recognition and orientation of substrate in that enzyme’s active site^10–12^. We found that many of the residues in CYP101A1 that are most perturbed upon substrate binding (and were subsequently found to be functionally important) lie in a conical region roughly anti-symmetric with the triangular lozenge shape of the P450 molecule (Fig. 8, top). We suspected that these observations might be general, and that other cytochromes P450 would show similar substrate-dependent displacements in the same regions as CYP101A1. In order to test this, we chose MycG, as it has desirable qualities as an NMR target (good stability and solubility, and is readily expressed in defined media) but is more similar to many eukaryotic P450s in that it binds a large substrate in a relatively open active site.

Figure 8 (bottom) shows that in fact many of the same regions are displaced upon substrate binding in MycG as in CYP101A1. The perturbations are larger in MycG, for which there are several possible reasons. Obviously, M-IV is much larger than camphor, and it is to be expected that substrate-dependent structural rearrangements would be larger in MycG than in CYP101A1. Secondly, the CYP101A1 structures were characterized in reduced form, which is diamagnetic and allowed resonance assignments to be made for amide NH groups much closer to the heme than in the predominantly high-spin oxidized camphor-bound form. The reduced form of CYP101A1 is less dynamic than the oxidized enzyme^6^, and likely to have a smaller conformational search space available to it, possibly attenuating the observed conformational shifts. MycG, on the other hand, is predominantly low spin in the oxidized form, even with M-IV bound^9^, and as the reduced MycG is relatively unstable, it was more convenient to work with the oxidized enzyme. The increased flexibility of the substrate-free MycG may also explain why some surface-exposed residues show consistent RDC violations (which were not observed in either forms of CYP101A1 or in substrate-bound MycG).

Nevertheless, the similarities in the regions that are displaced upon substrate binding in both MycG and CYP101A1 suggests that, despite their disparate biological niches and functions, structurally homologous regions of both enzymes fulfill similar roles, and we can begin to discern the outlines of a generally conserved mechanism for substrate binding and recognition in the P450 superfamily. We are currently investigating yet another member of the P450 superfamily, CYP2D6, an important human enzyme that is involved in drug activation and metabolism, in order to see if a P450 that is normally membrane-associated will also exhibit similar conformational changes upon binding of substrates.

## Experimental

### Sample preparation and NMR spectroscopy

MycG was expressed in appropriately isotopically labeled form and purified without substrate as detailed previously^9^. Samples of substrate-free MycG for measurement of RDCs were concentrated to 500 μM in appropriate NMR buffer (200 mM KCl, 50 mM KP_i_ pH 7.4, 80:20 H_2_O/D_2_O) and then diluted 1:1 with the appropriate alignment medium, (also in NMR buffer), either 10% v/v petaethylene glycol monododecyl ether (C12E5, Fluka) in 0.85 molar ratio with *n*-hexanol or 50 mg/mL *pf1* bacteriophage (Asla Bioteck, Riga Latvia). All NMR data was acquired on a Bruker Avance spectrometer (Brandeis University Landsman Research Facility) operating at 800.13 and 81.086 MHz for ^1^H and ^15^N, respectively. Residual dipolar couplings were measured using the TROSY/semi-TROSY difference method described by Weigelt^16^, as detailed previously^9^.

### RDC data analysis and structural ensemble generation

The processed TROSY/semi-TROSY data was analyzed using SPARKY^21^, and the measured RDCs translated into AMBER-readable format using macros available from *ambertools*. All molecular dynamics were performed using the AMBER 16 *sander* module installed on the XSEDE node Comet (UCSD)^18^. The simulation protocols used were identical to those published recently for substrate-bound MycG^9^.

### Structural analyses and alignments

Structural alignments and morphing between substrate-free and substrate bound forms of CYP101A1 and MycG were performed using Chimera^19^. Morphing was performed after alignment using a minimum of 40 corkscrew interpolation steps. Substrate and heme positions were not morphed, but maintained using the coordinates of the substrate-bound forms of each enzyme. As such, substrate fade-ins in videos are for visualization only.

### Supplementary Material available

Eleven videos animating morphs between substrate-free and substrate bound forms of MycG and CYP101A1, lists of RDCs used to restrain calculations of substrate-free MycG structures, PDB-format coordinates for REP2 with solvent removed.

## Electronic supplementary material

Video S1

Video S2

Video S3

Video S4

Video S5

Video S6

Video S7

Video S8

Video S9

Video S10

Video S11

Supplementary Information

Supplementary Dataset
