# Supplementary material for: Substrate recognition by two different P450s: Evidence for conserved roles in a common fold
Source: Sci Rep. 2017 Oct 19;7:13581. doi: 10.1038/s41598-017-14011-w (PMC5648816; doi:10.1038/s41598-017-14011-w)
Supplement: Supplementary file 12 — Supplementary Information [file 41598_2017_14011_MOESM12_ESM.pdf]

Supplementary Material for:

Substrate recognition by two different P450s: Evidence for conserved roles in a common fold

Drew R. Tietz,[a] Allison M. Colthart,[b] Susan Sondej Pochapsky[a] and Thomas C. Pochapsky[a, b,*]

a) Department of Chemistry and b) Rosenstiel Basic Medical Sciences Research Institute,

Brandeis University, MS 015, 415 South St., Waltham, MA 02453,

*To whom correspondence should be addressed: pochapsk@brandeis.edu

Video descriptions

Video S1. Global conformational changes upon binding of mycinamicin IV (M-IV, represented as spheres) to MycG as detected by RDC-directed soft annealing. Video is from the same perspective as Figure 2. Labels refer to secondary structural features named in Figure 2. Video cycles twice between unbound and M-IV-bound forms. Video generated using Chimera.[2]

Video S2. Conformational changes in the β-rich region of MycG upon binding of M-IV. M-IV is represented by line structure in the upper left corner of the video. The loop between the β1 strand and B helix (with Thr 43 labeled) is the least mobile, and appears to provide a pivot for the movement. Video starts with M-IV bound MycG and moves to the unbound form. Video generated using Chimera.[2]

Video S3. Changes in hydrogen bonding in the K' helix associated with the movement of the β-rich region shown in Video S2. Video cycles twice between substrate-free to M-IV bound MycG. Video generated using Chimera.[2]

Video S4. Close-up of conformational changes in the active site of MycG upon M-IV binding. Video cycles twice between substrate-free and M-IV bound MycG. M-IV is represented by spheres. Video generated using Chimera.[2]

Video S5. Hydrogen bond changes in the I helix near the O_2 binding site on the heme as a function of M-IV binding. Video moves from substrate-free and M-IV bound MycG. M-IV is represented by spheres. Video generated using Chimera.[2]

Video S6. View down the long axis of the I helix of MycG (from the N-terminal end), showing clockwise rotational motion as M-IV binds, and associated movements of the F and G helices. Video cycles twice between substrate-free and M-IV bound MycG. M-IV is represented by spheres. Video generated using Chimera.[2]

Video S7. Global conformational changes associated with binding of substrate camphor to CYP101A1 as detected by RDC-directed soft annealing. Substrate camphor is represented as spheres. Morph is between PDB entries 2LQD (substrate-free) and 2L8M (camphor-bound) CYP101A1. Molecule is presented in the same perspective as MycG in Video S1. Video generated using Chimera.[2]

Video S8. Conformational changes in the β-rich region of CYP101A1 associated with camphor binding. Pivot for the motion appears to be near Asn 59 (shown as sticks) on the turn between the two strands of the β1 sheet. Compare with Video S2 for MycG. Video generated using Chimera.[2]

Video S9. Close-up of changes in the CYP101A1 active site upon binding of camphor (shown as spheres). Video cycles twice between camphor-free and camphor-bound CYP101A1. Compare to Video S4 for MycG. Video generated using Chimera.[2]

Video S10. Changes in hydrogen bonding in the I helix of CYP101A1 upon camphor binding. Video cycles once between unbound and bound forms. Compare with Video S5 for MycG. Video generated using Chimera.[2]

Video S11. View down the long axis of the I helix of CYP101A1 (from the N-terminal end), showing counter-clockwise rotational motion as camphor binds, and associated movements of the C-D loop. Video cycles twice between substrate-free and camphor-bound CYP101A1. Camphor is represented by spheres. Compare with Video S6 for MycG. Video generated using Chimera.[2]

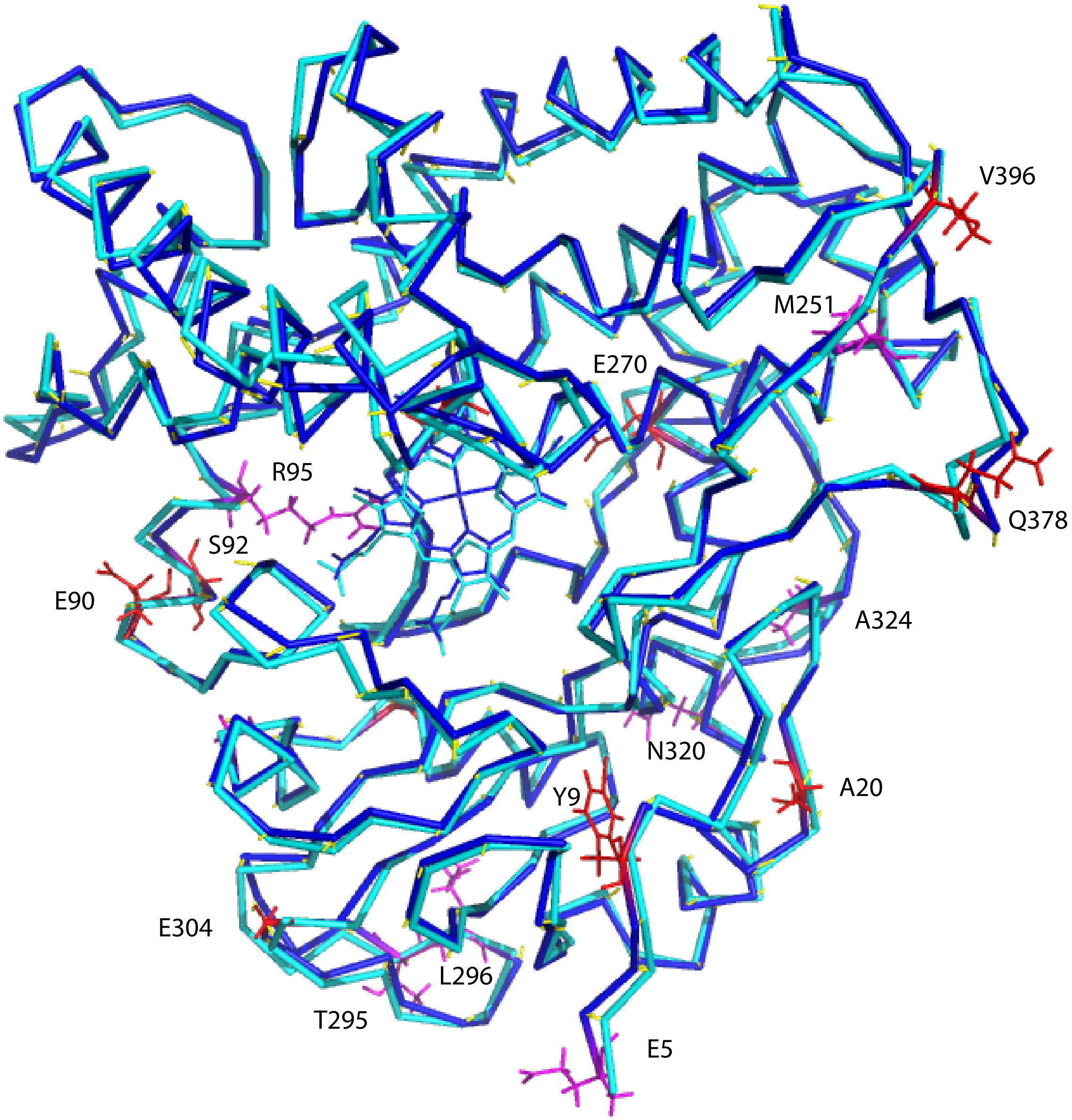

Figure S1. Superposition of REP2 (dark blue) backbone structure on a representative structure obtained using RDCs showing significant deviations from predicted values (labeled residues shown in red and purple). Structures are in approximately the same orientation as in Figure 2. See Table S1 and text for details. Figure generated using PyMOL.[1]

Table S1. RDCs showing consistent errors in global calculations. See text and Figure S1 for details.

pf1

R#	RDC (Hz)
1	-3
5	-11.0013
16	-13.40169
60	13.0026
91	9.4420215
247	9.1059669
291	10.2741567
292	6.8496003
316	8.20182
320	-18.1464609
392	-14.9379396

C12E5

R#	RDC (Hz)
5	-11.0013
16	-19.0026
53	-27.80403
86	-11.80143
88	-16.922262
231	9.00195
268	-18.20247
300	-9.6090738
374	-6.20052
392	-19.738719

Table S2. RDC restraints measured for substrate-free MycG with bacteriophage *pf1* used for calculations of REP2. Listed RDC value represents the lower limit given a +/- 3 Hz uncertainty.

R#	RDC (Hz)	R#	RDC (Hz)	R#	RDC (Hz)	R#	RDC (Hz)
		106	-3.80013	189	-6.8886318	309	-4.1921937
3	-3.0480078	107	-3.4960806	193	-6.20052	310	7.081638
4	-0.3115632	109	-6.20052	194	1.7927787	311	-3
7	0.20052	110	-5.000325	195	-8.2568541	312	-5.40039
10	1.80078	111	-4.60026	196	3.4490478	313	-2.1038544
11	-11.80143	112	-0.59961	199	-8.60091	314	7.3936887
12	1.80078	113	-7.2726942	202	-7.80078	315	-0.9436659
13	-6.6405915	119	-1.1116932	203	-7.6567566	317	5.6734092
14	-3.0640104	120	-6.5365746	204	8.041794	319	-10.20117
17	-2.19987	121	-1.39974	205	-7.00065	321	-12.5535522
18	-6.20052	122	2.0888268	211	0.20052	322	0.4485603
19	-10.441209	123	-2.8559766	212	-10.6172376	323	-2.3758986
20	-7.6567566	124	3.40104	213	-3.880143	324	-7.00065
21	-2.19987	125	7.8897693	215	-3	325	-4.5842574
22	-6.2645304	126	-10.20117	220	-4.60026	326	-7.80078
23	-5.40039	128	-5.2963731	221	3.9291258	328	-7.80078
24	4.1851674	129	-13.2496653	222	3.40104	335	-6.0244914
25	-4.7202795	130	-1.6157751	224	-3.80013	338	-5.40039
26	-12.7215795	132	-7.00065	226	-11.40234	345	-3
28	-11.80143	133	-3.6961131	227	-0.8956581	347	2.60091
29	-13.0496328	134	-3.1760286	231	-3	348	-4.60026
30	-10.4492103	135	-2.19987	234	-3.80013	355	-5.40039
31	-1.1757036	136	1.80078	236	5.0013	356	5.0013
32	1.9368021	142	-7.80078	239	-4.60026	359	-12.60156
35	3.321027	143	-9.2490153	240	5.80143	360	-10.20117
36	7.2096588	149	-10.20117	243	1.80078	361	-1.39974
37	-5.40039	152	-3.880143	246	-11.0013	362	-4.2322002
38	-9.3530322	153	-4.9043094	248	-1.2317127	363	-7.80078
39	-0.1915437	158	-7.00065	249	-10.20117	365	-15.00195
40	-4.4082288	159	-3.80013	251	-7.00065	366	-6.20052
41	-10.20117	161	-10.20117	252	-6.20052	367	2.60091
42	-9.7450959	162	-5.40039	253	6.60156	368	-11.5533897
43	-9.40104	170	-10.20117	254	-0.59961	369	-9.40104
44	-6.9686448	171	-12.60156	255	-7.80078	370	-0.9756711
45	-7.1766786	172	-2.0798505	256	4.8332727	372	-4.4002275
46	-6.2645304	173	6.9776211	257	-1.1837049	373	-4.4242314
47	-1.3757361	174	1.9688073	258	-0.6556191	374	-3
48	-1.7037894	175	-5.40039	259	0.20052	375	-0.59961
49	-3.7681248	182	2.60091	261	-6.5685798	377	-2.9279883
50	-13.5217095	183	-4.60026	262	-3.80013	378	-6.9126357
52	-3.0400065	184	-3	265	-8.60091	379	-5.6884368
53	-8.60091	185	-6.0965031	266	-11.4253689	380	-3.80013
54	-3.2720442	186	-8.4648879	267	-5.40039	382	-5.40039
55	4.20117	187	9.00195	268	-1.8718167	385	-0.1595385
56	-5.9524797	188	-1.079688	269	-4.60026	387	-2.3518947
57	2.2088463	189	-6.8886318	276	1.00065	388	-9.40104
58	-6.20052	193	-6.20052	277	-1.39974	391	-8.6569191
59	-6.0735518	194	1.7927787	278	-3.80013	393	-3.3920637
62	-2.3118882	195	-8.2568541	279	-12.60156	309	-4.1921937
63	7.2256614	196	3.4490478	280	-10.20117	310	7.081638
64	-4.2402015	199	-8.60091	284	-3	311	-3
65	2.60091	202	-7.80078	285	-5.2963731	312	-5.40039
66	1.9928112	203	-7.6567566	286	-3	313	-2.1038544
67	-5.40039	204	8.041794	287	3.40104	314	7.3936887
69	-0.2875593	205	-7.00065	288	-9.40104	315	-0.9436659
70	1.8327852	211	0.20052	289	5.0013	317	5.6734092
73	1.00065	212	-10.6172376	290	3.40104	319	-10.20117
77	-11.80143	213	-3.880143	293	0.20052	321	-12.5535522
79	-8.60091	215	-3	294	6.60156	322	0.4485603

80	-13.8497628	220	-4.60026	295	-9.40104	323	-2.3758986
82	-4.9763211	221	3.9291258	296	-4.60026	324	-7.00065
83	-7.6087488	222	3.40104	297	9.4660254	325	-4.5842574
86	1.80078	224	-3.80013	298	9.80208	326	-7.80078
88	-13.8657654	226	-11.40234	299	-2.0798505	328	-7.80078
90	0.20052	227	-0.8956581	300	-5.3363796	335	-6.0244914
92	-9.161001	231	-3	301	0.6085863	338	-5.40039
93	5.0013	234	-3.80013	303	-8.60091	345	-3
97	1.80078	236	5.0013	304	-10.20117	347	2.60091
103	-3.80013	239	-4.60026	305	-11.80143	348	-4.60026
104	-4.840299	240	5.80143	306	-3	355	-5.40039
105	-2.8959831	106	-3.80013	307	-1.3597335	356	5.0013

Table S3. RDC restraints measured for substrate-free MycG with nematic liquid crystal medium C12E5/hexanol used for calculations of REP2. Listed RDC value represents the lower limit given a +/- 3 Hz uncertainty.

R#	RDC (Hz)	R#	RDC (Hz)	R#	RDC (Hz)	R#	RDC (Hz)
1	-3	119	-5.7844524	259	-2.19987	378	-4.1921937
3	-3.0720117	120	2.8009425	261	0.712603	380	7.081638
4	-3.80013	121	1.00065	262	5.0013	382	-3
7	-3	122	6.9936237	265	-2.19987	385	-5.40039
10	-3.80013	123	-5.40039	266	-12.3535	387	-2.1038544
11	-16.8582516	125	9.8100813	267	-0.59961	388	7.3936887
12	-7.3046994	126	-9.40104	269	-4.53624	391	-0.9436659
13	-3.1440234	128	-4.3762236	276	-0.59961	393	5.6734092
14	-7.480728	129	-18.026441	277	-10.2011		
17	6.60156	130	-5.8964706	278	2.60091		
18	2.60091	132	-11.80143	279	-10.2011		
19	-9.3130257	133	-12.497543	280	-16.6022		
20	-7.2326877	134	-11.489379	284	-2.19987		
21	-6.0724992	135	-2.19987	286	-2.19987		
22	-4.6322652	136	1.80078	287	1.00065		
23	0.20052	142	1.3847124	288	2.60091		
24	0.1605135	143	-7.6567566	289	-10.8972		
25	-6.9526422	149	-5.40039	290	9.80208		
26	-17.40234	152	-8.4728892	291	9.506031		
28	-18.20247	153	-6.8646279	292	10.58620		
29	-16.6902243	158	-18.20247	293	4.20117		
30	-13.8097563	159	-2.19987	294	5.681410		
31	-9.6810855	161	-18.20247	295	1.80078		
32	-6.2565291	162	-16.60221	296	-10.3611		
35	6.041469	170	-10.20117	297	6.577556		
36	10.6742217	172	-2.6479428	298	8.20182		
38	-2.1518622	173	5.0573091	299	-3.99216		
39	0.840624	174	2.1448359	301	4.289184		
40	-7.1526747	175	5.0013	303	-10.1051		
41	-20.4828405	182	7.40169	304	-17.4023		
43	-20.60286	183	0.20052	305	-13.4016		
44	-11.0013	184	5.0013	306	-11.8014		
45	-5.9924862	185	-10.241176	307	-2.19987		
46	-16.3861749	186	-5.2643679	308	5.0013		
47	-5.6324277	187	7.40169	309	-7.00065		
48	-11.3853624	188	1.8407865	310	1.136672		
49	-11.0013	189	-6.7446084	311	-3.80013		
50	-13.40169	193	-4.60026	312	3.40104		
52	-10.6892493	194	1.8967956	313	-10.9052		
54	-4.5602535	195	-8.3208645	314	-0.77563		
55	12.20247	196	-5.6564316	316	6.60156		
56	-6.20052	199	-7.00065	317	13.40266		
57	0.0484953	202	-6.20052	319	-15.8020		
58	-8.60091	203	-9.0409815	320	-12.6015		
59	-6.9857	204	-1.5997725	321	-16.4181		
62	0.0885018	211	-8.4248814	322	0.496568		
63	-2.8079688	212	-10.20117	323	0.408553		
64	-6.5765811	213	-6.9126357	324	-1.88781		
65	1.80078	215	7.40169	325	3.881118		
66	1.80078	220	-6.20052	326	-13.4016		
67	-17.40234	221	-7.72076	327	0.20052		
69	-0.7196295	222	-2.19987	328	-2.19987		
73	-9.40104	224	-0.59961	334	6.60156		
79	-4.120182	227	0.20052	335	-6.29653		
80	-10.2651804	234	-4.60026	338	-2.19987		
82	3.5930712	236	5.0013	340	-6.20052		
83	-0.59961	239	2.60091	345	-0.59961		
90	-7.80078	240	4.20117	347	-8.60091		

91	-0.9596685	243	-7.80078	361	0.20052		
92	-5.2723692	246	0.20052	362	-4.60026		
97	-3	247	-1.0156776	363	-1.39974		
103	6.60156	248	2.7529347	365	-20.6028		
104	-6.1285083	249	-2.19987	366	-4.84830		
105	-9.3370296	251	-3	367	-3		
106	-15.00195	252	4.20117	368	0.520572		
107	-4.680273	253	-5.40039	369	-9.72109		
109	-7.2726942	254	-13.40169	370	-0.75163		
110	-2.599935	255	-11.80143	372	5.465375		
111	7.40169	256	2.8649529	373	-10.4412		
112	6.60156	257	-11.529385	375	-7.80078		
113	-8.60091	258	-1.8478128	377	-1.39974		

Table S4. Principal components of optimized alignment tensors used in calculations of REP2.

pf1 tensor	**1**	**2**	**3**
**1**	4.1814	-29.857	-21.636
**2**	-29.857	-14.102	-2.015
**3**	-21.636	-2.015	18.286

C12 tensor	**1**	**2**	**3**
**1**	1.619	-25.364	-2.433
**2**	-25.364	-1.096	-7.644
**3**	-2.433	-7.644	0.523

1 The PyMOL Molecular Graphics System v. 1.7.4 (2015).
2 Pettersen, E. F. *et al.* UCSF chimera - A visualization system for exploratory research and analysis. *Journal of Computational Chemistry* **25**, 1605-1612, doi:10.1002/jcc.20084 (2004).
